# Severe insulin-requiring diabetes in a noradrenergic giant pheochromocytoma: a secretory-metabolic phenotype paradox

**DOI:** 10.1210/jcemcr/luag323

**Published:** 2026-10-22

**Authors:** Luiz F Viola, Asma Guedioura, Josiane Neves Coelho, Adriana Santi, Catherine Campinos

**Affiliations:** Department of Endocrinology, Diabetes and Nutrition, NOVO Hospital, Pontoise 95300, France; Department of Endocrinology, Diabetes and Nutrition, NOVO Hospital, Pontoise 95300, France; Faculty of Health Sciences, Federal University of Rondonópolis, Rondonópolis 78736-900, Brazil; Faculty of Health Sciences, Federal University of Rondonópolis, Rondonópolis 78736-900, Brazil; Department of Endocrinology, Diabetes and Nutrition, NOVO Hospital, Pontoise 95300, France

**Keywords:** pheochromocytoma, noradrenergic, diabetes mellitus, catecholamines, adrenalectomy, β-cell dysfunction

## Abstract

Pheochromocytoma is a rare catecholamine-secreting neuroendocrine tumor associated with hypertension and dysglycemia in 20% to 40% of cases. Insulin-requiring diabetes has been reported in tumors with an adrenergic profile, owing to epinephrine-mediated inhibition of β-cell secretion. A 37-year-old man with a normal body mass index and an 18-year history of insulin-requiring diabetes and hypertension was found to have a right adrenal mass measuring 10.1 × 8.0 × 6.7 cm. Biochemical evaluation showed a noradrenergic profile, with urinary normetanephrine greater than 5000 μg/24 hours (SI: >26 400 nmol/day; reference range, 70-538 μg/24 hours [SI: 370-2841 nmol/day]) and normal urinary metanephrine. Right adrenalectomy and histopathology confirmed an intra-adrenal pheochromocytoma. After surgery, insulin was discontinued, and glycated hemoglobin remained below 6.1% (SI: 41-43 mmol/mol) without therapy. A preoperative insulin requirement of 0.83 units/kg/day, normal C-peptide, and negative glutamic acid decarboxylase antibodies supported a mixed and reversible mechanism involving catecholamine-induced insulin resistance and β-cell suppression. Severe postoperative hypoglycemia was attributed to reversal of catecholamine excess. This case challenges the association of severe diabetes with adrenergic pheochromocytomas and suggests that catecholamine burden and exposure duration may be more important determinants of metabolic severity than secretory profile alone, after prolonged exposure to high catecholamine concentrations.

## Introduction

Pheochromocytoma is a rare neuroendocrine tumor arising from the chromaffin cells of the adrenal medulla, characterized by excessive catecholamine secretion. Its annual incidence is estimated at approximately 0.8 cases per 100 000 individuals, although autopsy series suggest a substantial proportion of undiagnosed cases [1, 2].

Beyond its classic cardiovascular manifestations, pheochromocytoma is associated with significant metabolic disturbances, notably glucose intolerance or diabetes mellitus observed in 20% to 40% of patients [3, 4]. Hyperglycemia results from complex mechanisms involving inhibition of insulin secretion via activation of α2-adrenergic receptors on pancreatic β-cells, increased hepatic glucose production, and induction of peripheral insulin resistance [5].

The biochemical profile of pheochromocytomas distinguishes tumors with an adrenergic predominance (epinephrine secretion) from those with a noradrenergic predominance (norepinephrine secretion), a distinction that partly reflects the molecular subgroups (clusters) described in the tumorigenesis of these neoplasms [6, 7]. Adrenergic tumors have been more frequently associated with severe forms of diabetes due to the direct inhibitory effect of epinephrine on insulin secretion. We report the case of a giant pheochromocytoma with a noradrenergic biochemical profile that mimicked insulin-requiring type 1 diabetes mellitus (T1DM) for 18 years, with complete remission following adrenalectomy. This observation challenges the classical correlation between secretory phenotype and metabolic expression, and suggests that total catecholaminergic burden and duration of exposure may play a determining role in the severity of metabolic manifestations.

## Case presentation

A 37-year-old male with a normal body mass index (BMI 23.7 kg/m^2^; weight 75 kg, height 1.78 m) was referred to the endocrinology department following right adrenalectomy for resection of a large adrenal mass. His medical history was notable for T1DM, diagnosed 18 years prior at the time of initial presentation with dizziness, polyuria, diaphoresis, significant weight loss, and recurrent syncopal episodes. Despite intensive insulin therapy with regular follow-up, glycemic control progressively deteriorated over the ensuing years. At referral, his insulin regimen comprised intermediate-acting neutral protamine Hagedorn insulin, 34 units/day (16 units in the morning and 18 units in the evening) and short-acting (regular) insulin 28 units/day (8-10-10 units), a total daily dose of 62 units/day, corresponding to 0.83 units/kg/day. Six years after the diabetes diagnosis, arterial hypertension was identified and partially managed with losartan 50 mg and amlodipine 5 mg twice daily. His family history was significant for type 2 diabetes mellitus in his father and hypertension in both his father and brother.

## Diagnostic assessment

Eighteen years after the initial diabetes diagnosis, the patient presented with abdominal pain. Urinalysis revealed hematuria and proteinuria, prompting abdominal ultrasonography, which demonstrated a right adrenal mass with irregular contours and internal vascularity on color Doppler imaging. Subsequent contrast-enhanced abdominal computed tomography (CT) identified a right adrenal tumor measuring 10.1 × 8.0 × 6.7 cm, featuring central necrotic and liquefied areas, with close anatomical relationship to the adjacent liver and right kidney (Fig. 1A and 1B).

**Figure 1:**
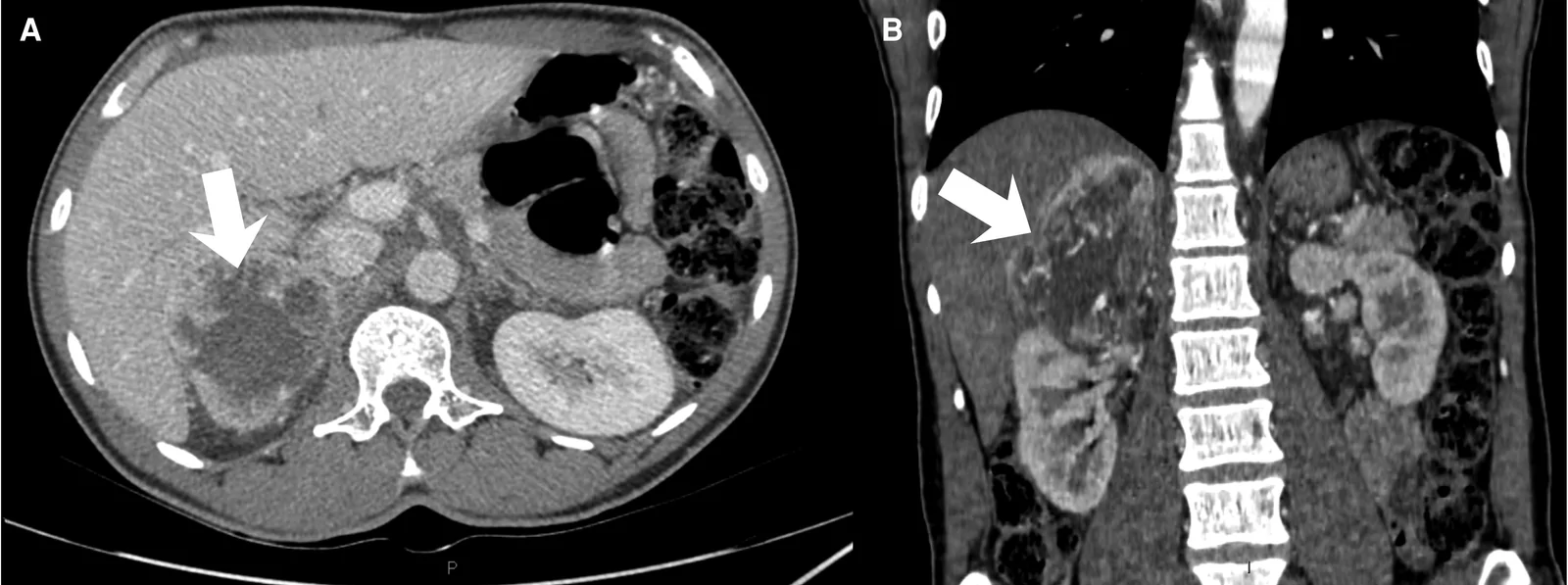
Abdominal computed tomography scan in axial (A) and coronal (B) planes showing a necrotic giant pheochromocytoma overlying the right kidney, measuring 10.1 × 8.0 × 6.7 cm.

Biochemical evaluation revealed a glycated hemoglobin (HbA1c) of 8.9% (SI: 74 mmol/mol; reference range, 4.0-5.6% [SI: 20-38 mmol/mol]). Urinary catecholamine metabolite profiling demonstrated a markedly noradrenergic secretory pattern. Urinary normetanephrine was greater than 5000 μg/24 hours (SI: >26 400 nmol/day; reference range, 70-538 μg/24 hours [SI: 370-2841 nmol/day]), whereas urinary metanephrine remained within the reference range at 97.5 μg/24 hours (SI: 515 nmol/day; reference range, 66-124 μg/24 hours [SI: 349-655 nmol/day]). Urinary norepinephrine was markedly elevated at 2062 μg/24 hours (SI: 12 213 nmol/day; reference range, <97 μg/24 hours [SI: <574 nmol/day]), confirming a predominantly noradrenergic biochemical phenotype. Laboratory values before and after adrenalectomy are summarized in Table 1.

**Table 1:** Laboratory values before and after right adrenalectomy

Variable	Preoperative	Postoperative	Reference range
HbA1c	8.9% (74 mmol/mol)	5.9-6.1% (41-43 mmol/mol)	4.0-5.6% (20-38 mmol/mol)
Fasting plasma glucose	205 mg/dL (11.4 mmol/L)	121.5 mg/dL (6.75 mmol/L)	70-99 mg/dL (3.9-5.5 mmol/L)
Urinary normetanephrine	>5000 μg/24 hours (>26 400 nmol/24 hours)	Not available	70-538 μg/24 hours (370-2841 nmol/24 hours)
Urinary metanephrine	97.5 μg/24 hours (515 nmol/24 hours)	Not available	66-124 (349-655 nmol/24 hours)
Plasma normetanephrine	Not available	104 pg/mL (568 pmol/L)	<196 pg/mL (<1070 pmol/L)
Plasma metanephrine	Not available	24 pg/mL (122 pmol/L)	<65 (<330 pmol/L)
Urinary norepinephrine	2062 μg/24 hours (12 213 nmol/24 hours)	Not available	<97 μg/24 hours (<574 nmol/24 hours)
Fasting C-peptide	Not available	2.58 ng/mL (0.86 nmol/L)	1.1-4.4 ng/mL (0.37-1.47 nmol/L)

Abbreviations: anti-GAD, antiglutamic acid decarboxylase; HbA1c, glycated hemoglobin.

Conventional units are given first, with Système International (SI) units in parentheses.

## Treatment

The patient underwent radical right adrenalectomy and nephrectomy. Nephrectomy was necessitated by the intimate anatomical relationship between the tumor and the right kidney, with intraoperative findings precluding organ preservation. Notably, no preoperative endocrinology consultation was obtained, and appropriate perioperative pheochromocytoma management, including alpha-adrenergic blockade and hemodynamic optimization, was not performed prior to surgery. In the immediate postoperative period, the patient experienced life-threatening episodes of hypoglycemia and hemodynamic instability.

Histopathological examination of the surgical specimen revealed a nodular lesion measuring 10.5 × 6.5 × 5.2 cm and weighing 195 g that occupied almost the entire right adrenal gland, with a free capsular margin and a Zellballen architecture. Immunohistochemical staining was positive for chromogranin A and synaptophysin and negative for S-100 and Melan-A in the tumor cells, with S-100 positivity confined to adjacent brown adipose tissue, confirming the diagnosis of an intra-adrenal pheochromocytoma of the right adrenal gland; in the 2022 World Health Organization classification the term pheochromocytoma denotes an intra-adrenal paraganglioma [8]. Germline genetic testing (including *SDHB*, *SDHD*, and *VHL*) was not performed, representing a limitation of this case.

## Outcome and follow-up

At 1 month postoperatively, arterial hypertension had resolved without antihypertensive therapy. However, the patient continued to experience recurrent hypoglycemic episodes despite progressive reduction of his insulin dose. Upon referral to our endocrine service, laboratory reassessment revealed a fasting plasma glucose of 121.5 mg/dL (SI: 6.75 mmol/L; reference range, 70-99 mg/dL [SI: 3.9-5.5 mmol/L]) and a markedly improved HbA1c of 6.1% (SI: 43 mmol/mol). Plasma fractionated metanephrines had normalized (metanephrine, 24 pg/mL [SI: 122 pmol/L]; normetanephrine, 104 pg/mL [SI: 568 pmol/L]). Further investigations showed a normal fasting C-peptide level of 2.58 ng/mL (SI: 0.86 nmol/L) and negative antiglutamic acid decarboxylase (anti-GAD) antibodies, effectively excluding autoimmune β-cell destruction as the underlying mechanism.

In light of these findings, insulin therapy was discontinued. Over subsequent follow-up visits, the patient maintained an HbA1c between 5.9% and 6.1% (SI: 41-43 mmol/mol) on no antidiabetic medication, confirming complete, and durable remission of diabetes following tumor resection.

## Discussion

Disorders of glucose metabolism associated with pheochromocytoma are well documented, but their severity appears to depend on the tumor's biochemical profile [4]. Classically, tumors with an adrenergic phenotype are more frequently associated with overt diabetes due to inhibition of insulin secretion via activation of α2-adrenergic receptors on pancreatic β-cells [1, 4, 9]. Conversely, noradrenergic phenotype tumors are traditionally described as essentially vasopressive, with less pronounced metabolic impact [2, 3]. Our observation challenges this dichotomy.

This patient presented with insulin-requiring diabetes diagnosed at age 19, evolving for 18 years before the discovery of a giant pheochromocytoma with a clearly noradrenergic biochemical profile (major elevation of normetanephrine without significant excess of metanephrine). The severity and duration of hyperglycemia, mimicking T1DM, are unexpected in the context of a predominantly noradrenergic tumor.

The noradrenergic profile of a giant intra-adrenal tumor is mechanistically coherent rather than paradoxical. Conversion of norepinephrine to epinephrine depends on phenylethanolamine N-methyltransferase (PNMT), whose expression in the adrenal medulla is induced by the high local glucocorticoid concentration supplied by the adrenal cortical portal circulation. In large adrenal tumors this cortical glucocorticoid gradient is disrupted, PNMT induction is lost, and secretion shifts toward norepinephrine and its metabolite normetanephrine, so that even an intra-adrenal pheochromocytoma may become predominantly noradrenergic [6, 8]. This mechanism reconciles the intra-adrenal origin confirmed at histopathology with the observed noradrenergic secretory phenotype.

Current pathophysiological models primarily attribute the diabetogenic effect of pheochromocytomas to epinephrine, due to its direct inhibitory action on the early phase of insulin secretion [4]. However, norepinephrine shares affinity for α2 receptors and may, in cases of massive chronic exposure, induce: prolonged inhibition of β-cell function; peripheral insulin resistance; persistent stimulation of hepatic gluconeogenesis and glycogenolysis; and lipolysis with elevation of free fatty acids, further aggravating insulin resistance [5].

In our patient the preoperative total insulin requirement (62 units/day; 0.83 units/kg/day) in the setting of a normal BMI was within the usual range for type 1 diabetes and was not markedly elevated, arguing against a phenotype driven predominantly by insulin resistance. Taken together with the complete postoperative remission, a fasting C-peptide within the reference range, and negative anti-GAD antibodies, these findings support a mixed mechanism, combining catecholamine-induced insulin resistance with functional β-cell suppression, the latter accounting for the preserved β-cell mass and the reversibility after resection [4, 5].

The tumor size (>10 cm) and extremely elevated catecholaminergic burden observed in our patient suggest that total hormonal load and duration of chronic exposure may be more important determinants of the metabolic phenotype than the qualitative nature of the secretory profile alone. The normalization of glycemic control following tumor resection, with complete discontinuation of insulin and maintenance of HbA1c < 6.1% (SI: <43 mmol/mol), confirms the secondary and reversible nature of the hyperglycemia. Previous studies have demonstrated significant improvement in insulin sensitivity after adrenalectomy [4, 10], but complete reversibility after 18 years of insulin therapy remains remarkable and underscores the exclusively functional nature of β-cell impairment in this case.

The severe hypoglycemia observed in the immediate postoperative period is likely explained by the abrupt release of β-cell inhibition, rapid restoration of insulin sensitivity, a possible chronic adaptive reduction in glucagon secretion [11], and continuation of exogenous insulin in the absence of the prior catecholamine excess. This case illustrates the critical importance of preoperative endocrinology consultation and perioperative alpha-adrenergic blockade, which were unfortunately not implemented in this patient. Elevated urinary metanephrine concentrations and tumor size have been identified as risk factors for postoperative hypoglycemia [12].

The biochemical phenotype of pheochromocytomas is often correlated with molecular clusters [6]. Noradrenergic tumors are generally associated with cluster 1 (pseudohypoxia: *VHL*, *SDHx*), while adrenergic tumors more commonly belong to cluster 2 (*RET*, *NF1*). The absence of germline genetic testing in our case is a limitation; given the tumor's noradrenergic profile and large size, testing for *SDHB*, *SDHD*, and *VHL* mutations would have been clinically indicated [13]. Nonetheless, the observed clinical presentation suggests that the secretory-metabolic phenotype correlation may be more complex than assumed, and that certain large noradrenergic tumors can induce a prolonged functional insulinopenic phenotype without irreversible β-cell destruction.

## Learning points

- Noradrenergic pheochromocytomas can cause severe, insulin-requiring diabetes. Although adrenergic tumors are classically associated with diabetic phenotypes, chronic massive norepinephrine exposure can induce prolonged functional β-cell suppression mimicking type 1 diabetes mellitus.
- Total catecholaminergic burden and duration of exposure may matter more than the secretory phenotype. Tumor size and chronicity of catecholamine excess may be more critical determinants of metabolic severity than the adrenergic vs noradrenergic biochemical profile alone.
- In a young patient with atypical, insulin-requiring diabetes (eg, preserved C-peptide and negative islet autoimmunity) associated with difficult-to-control hypertension, a secondary and potentially curable cause such as pheochromocytoma should be considered in line with current guidelines.
- β-Cell dysfunction in pheochromocytoma is functional and reversible. Complete diabetes remission after adrenalectomy, confirmed by normalization of C-peptide and negative anti-GAD antibodies, underscores the importance of timely diagnosis to avoid years of unnecessary insulin therapy.
- Perioperative alpha-adrenergic blockade is mandatory regardless of biochemical phenotype. Failure to prepare patients appropriately before surgery risks life-threatening hemodynamic instability and postoperative hypoglycemia.

## Contributors

All authors made individual contributions to authorship. L.F.V.: patient care, conception, data collection, literature review, drafting, and critical revision of the manuscript. A.G.: literature review, drafting, and critical revision of the manuscript. J.N.C. and A.S.: data collection and critical revision. C.C.: supervision, critical revision, and final approval. All authors reviewed and approved the final draft.

## Data Availability

Original data generated and analyzed during this study are included in this published article.
